# Retraction: MiR-543 Inhibits the Migration and Epithelial-To-Mesenchymal Transition of TGF-β-Treated Endometrial Stromal Cells via the MAPK and Wnt/β-Catenin Signaling Pathways

**DOI:** 10.3389/pore.2023.1611110

**Published:** 2023-05-25

**Authors:** Pathology and Oncology Research Editorial Office

Following publication, concerns were raised regarding data misrepresentation. In particular, with the Western blot in Figure 4A, which also appears in Zhang et al. [1]. Following provision of raw data by the authors, the Editor in Chief concluded that the article’s conclusions and assertions were not sufficiently supported by the findings from the material provided; therefore, the article has been retracted.
